# Routes to Diagnosis in Lung Cancer—Do Socio-Demographics Matter? An English Population-Based Study

**DOI:** 10.3390/cancers17111874

**Published:** 2025-06-03

**Authors:** Ruth P. Norris, Elizabeth Fuller, Alastair Greystoke, Adam Todd, Linda Sharp

**Affiliations:** 1 Population Health Sciences Institute, Newcastle University Centre for Cancer, Newcastle-upon-Tyne NE1 7RU, UK; ruth.norris@newcastle.ac.uk; 2 Cardiothoracic Directorate, Freeman Hospital, Newcastle Hospital Trust, Newcastle-upon-Tyne NE7 7DN, UK; liz.fuller@nhs.net; 3 Northern Centre for Cancer, Freeman Hospital, Newcastle Hospital Trust, Newcastle-upon-Tyne NE7 7DN, UK; alastair.greystoke@newcastle.ac.uk; 4 School of Pharmacy, Newcastle University, Newcastle-upon-Tyne NE1 7RU, UK; adam.todd@newcastle.ac.uk

**Keywords:** early diagnosis, emergencies, lung neoplasms, primary healthcare, socio-economic factors

## Abstract

**Objectives:** Survival from lung cancer is worse in the UK than in some other countries, with late stage at diagnosis implicated in poor prognosis. The route and referral urgency by which patients obtain a diagnosis influence outcomes. This study investigated whether socio-demographic factors are associated with lung cancer routes to diagnosis in England. **Materials and Methods:** A total of 181,763 primary invasive lung cancers (ICD-10 C34.0-C34.9) diagnosed from 1 January 2012 to 31 December 2016 were abstracted from the English National Cancer Registration Database. Multivariable logistic regression was used to examine associations between patients’ socio-demographic characteristics and likelihood (adjusted odds ratios) of (i) emergency presentation versus all primary care-initiated routes and (ii) urgent (“two-week wait”/2WW) versus standard primary care-initiated referral. Models included the following factors: deprivation quintile of area of residence at diagnosis (IMD income domain); sex; age; ethnic group; rural/urban residence; and (in the emergency model) region. **Results:** Socio-demographic variations in diagnosis routes were observed. Patients presenting as emergencies (35.2%) were more likely to be 80 years of age or older, female, of non-White ethnicity, and resident in areas of greater deprivation or the London region. In contrast, 2WW patients (28.3%) were more likely to be aged between 50 and 69 years old, of White ethnicity, and resident in an area of greater deprivation or resident outside of an urban centre; diagnosis through 2WW did not vary by sex. **Conclusions:** Routes to diagnosis are subject to distinct socio-demographic patterning. Action is needed to ensure that new referral guidelines and lung cancer screening roll-out do not widen socio-demographic inequalities in diagnosis.

## Simple Summary

Survival from lung cancer is worse in the UK than in some other high-income countries. A patient’s journey to a lung cancer diagnosis and the urgency of their referral for investigation (called their “route to diagnosis”) may affect how long they survive. We investigated whether route to diagnosis for lung cancer patients in England varies by socio-demographic characteristics. More than one third of lung cancer patients in England were diagnosed after presenting as an emergency. Being diagnosed as an emergency was more common in patients who were 80 years or older, female, belonged to non-White ethnic groups or lived in deprived areas. Just under 30% of patients were diagnosed following an urgent referral from their family doctor. These patients were more likely to be White and live in a deprived area or outside an urban centre. Improved referral guidelines are needed to address these differences in diagnostic routes.

## 1. Introduction

Long-term lung cancer prognosis remains poor, with late-stage diagnosis limiting treatment options [1,2]. Whilst survival is improving, the UK lags behind other high-income countries such as Canada, Denmark, and Norway, and, in a recent study of seven countries, the UK was found to have the lowest 5-year net survival [3]. One potential explanation for this is the fact that fewer people are diagnosed at an early stage [1].

Early lung cancer is often asymptomatic; symptoms may be non-specific and masked by, or attributed to, smoking-related ailments, which can delay patient help-seeking [4,5]. Lung cancer screening has only recently begun to be widely implemented; in the UK, targeted screening (low-dose computed tomography (CT) scan referral for smokers/former smokers aged 55–75 that have a Prostate Lung Colorectal Ovarian model risk ≥1.51% and Liverpool Lung Project risk model score ≥2.00%) began phased introduction from June 2023 following the success of the 2019 Targeted Lung Health Check pilot [6,7]. Currently, there is no blood test to detect lung cancer, though trials are ongoing (e.g., the SUMMIT study) [7]. Consequently, many lung cancers are diagnosed at a late stage and/or present as emergencies, and their prognosis is poor [2]. Prioritising earlier detection through clear referral pathways is necessary to help improve outcomes.

In England, the urgent care pathway (two-week wait; “2WW”) was introduced in 2000 to shorten the time to diagnosis (referral of suspected cancer to secondary care specialists for review within 2 weeks of presentation), enable earlier diagnosis, offer improved treatment choices, and increase survival [8]. This pathway did increase cancer referrals [9], but, over time, the percentage of 2WW referrals leading to a cancer diagnosis has declined [10]. In October 2023, 2WW was removed as a national standard (though data collection still occurs) and replaced with the Faster Diagnosis Standard (FDS), which states that patients referred by their general practitioner (GP) as a Suspected Cancer Referral should have a diagnosis or ruling-out of cancer within 28 days of referral [11]. Implementation of a best practice timed diagnosis pathway has also drawn on earlier recommendations from the National Optimal Lung Cancer Pathway (August 2017) [12].

Changes to referral pathways and targets have occurred against a background of inequalities across the lung cancer pathway, from symptom recognition to death. For example, incidence is socio-economically patterned [13]; time to diagnosis differs by ethnicity [14]; receipt of traditional and newer treatments varies by socio-economic status [15]; care experiences are worse in minority groups [16]; and rural residents have a higher risk of early death [17]. People residing in areas of high disadvantage are less likely to attend screening, more likely to smoke, may have poorer symptom recognition, and face practical, financial, or social barriers to obtaining care [18,19,20]. These factors, in turn, may influence whether, how rapidly, and through which route an individual with lung cancer obtains a diagnosis.

Previous studies exploring cancer routes to diagnosis suggest that there is demographic patterning by age [21], ethnicity [16], sex, and deprivation [22]. However, whilst there has been some exploration of routes to diagnosis within an English and a wider European context [23], studies with a lung cancer focus are rarer and have largely been descriptive. We set out to extend past work by undertaking a population-based study to investigate socio-demographic factors specifically associated with lung cancer routes to diagnosis in England. We had two distinct research questions. The first was to investigate socio-demographic factors related to diagnosis as an emergency (versus through primary care-initiated routes). The second considered only primary-care-initiated routes and sought to determine which socio-demographic factors are associated with diagnosis through 2WW versus any standard route.

## 2. Materials and Methods

### 2.1. Setting and Data Sources

In England, all residents are entitled to cancer care from the National Health Service (NHS), which is provided free at the point of delivery. This includes access to primary care services where GPs act as gatekeepers to hospital services, including diagnostic investigations and cancer treatment. The exceptions to this are accident and emergency (and related urgent care) services, which are located within hospitals but can be accessed directly without first needing to see a GP. This structure provides the rationale for having two distinct research questions in the current study.

Population-based data on all primary invasive lung cancers (International Classification of Diseases [ICD]-10 C34.0-C34.9) diagnosed between 1 January 2012 and 31 December 2017 (the most recent period for which complete data were available at time of data request) were abstracted from the National Cancer Registry Database (NCRD), the national cancer registry for England. The NCRD collects information from healthcare providers on all people in England diagnosed with malignant or premalignant neoplasms (approximately 300,000 tumour records annually across 162 healthcare providers) [24].

“Routes to diagnosis” (RTD) refers to a sequence of interactions between the patient and the healthcare system that leads to a cancer diagnosis [25]. An RTD variable was provided in the dataset requested. It was derived based on interactions recorded in several national, routine, health service datasets, namely: the NCRD, Hospital Episode Statistics, Cancer Waiting Times, and Cancer Screening Programme data. The variable has eight categories: standard GP referral; 2WW; emergency presentation; other outpatient; screen-detected (not relevant in this study); inpatient elective; death certificate only (DCO); and unknown. The unknown group is a result of the fact that not all of the health service interactions a patient might have on the way to diagnosis (e.g., general practitioner appointments) are recorded on national NHS datasets.

Additional data fields accessed from the NCRD included all those that capture patient socio-demographics (i.e., deprivation category of area of residence measured using the index of multiple deprivation (IMD), a widely used proxy for socio-economic status (SES); sex; age group at diagnosis; ethnic group; rural/urban residence; government region) and clinical variables (stage at diagnosis (TNM summary stage) [26]; tumour histology classification (ICD-02 and ICD-03) [27]; presence of multiple tumours (i.e., whether the individual had previously been diagnosed with another cancer); number of comorbidities (type of comorbidities was not included); whether discussed at multidisciplinary team (MDT) meeting; and year of diagnosis.

### 2.2. Population

**Figure 1:**
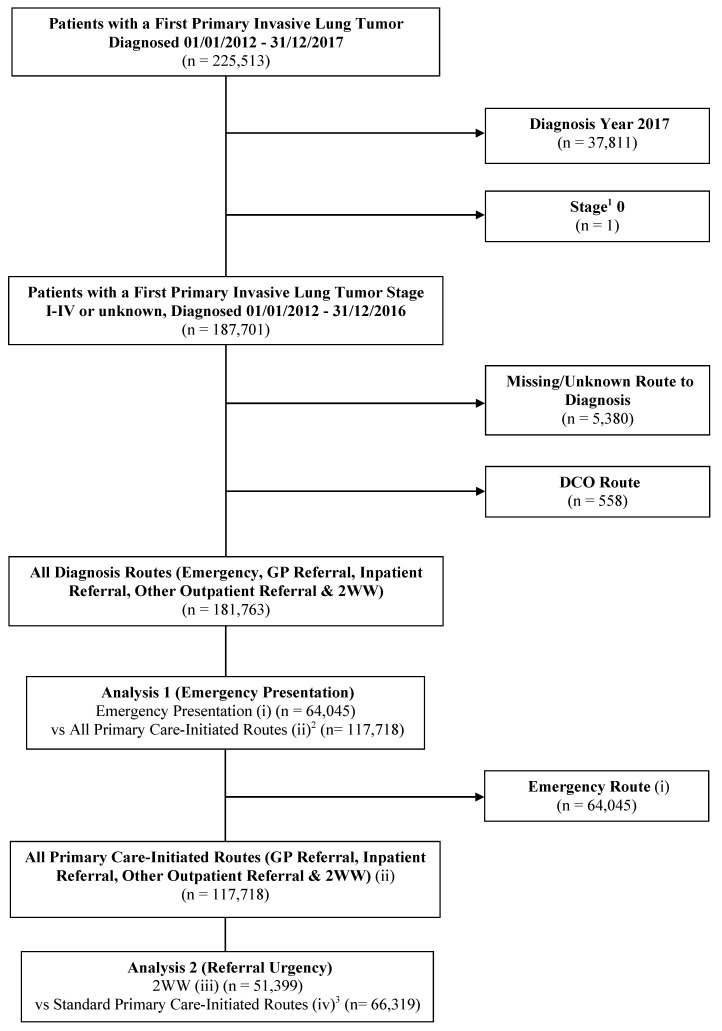
Flow diagram depicting the analytic cohorts for Analyses 1 and 2. ^1^ refers to stage at diagnosis. ^2^ refers to standard GP referral, inpatient referral, other outpatient referral, and 2WW (urgent care referral via a GP) diagnosis routes. ^3^ refers to standard GP referral, inpatient referral, and other outpatient referral diagnosis routes. Categories (i), (ii), (iii), and (iv) refer to the route to diagnosis sub-categories used in the analysis (see Section 2.3). Abbreviations: DCO: Death certificate only; GP: General practitioner; vs: Versus; 2WW: Two-week wait.

Where patients had multiple primary lung tumour registrations, we retained the following for analysis: (1) the earliest diagnosed tumour; (2) the most advanced staged tumour at diagnosis; (3) the most specific ICD coded tumour (ICD-10 C34.0-C34.8); or (4) the first tumour entry. Patients diagnosed in 2017 (*n* = 37,811) were excluded as the RTD variable only covered 2012–2016. We also excluded the following: stage 0 cancers (*n* = 1); unknown route to diagnosis (*n* = 5380); and DCO diagnoses (*n* = 558). This left an analytical population of 181,763 patients (Figure 1). Supplementary Table S1 summarises the characteristics of the patients excluded on the basis of the RTD being DCO or unknown (*n* = 5938).

### 2.3. Outcome Variables

**Figure 2:**
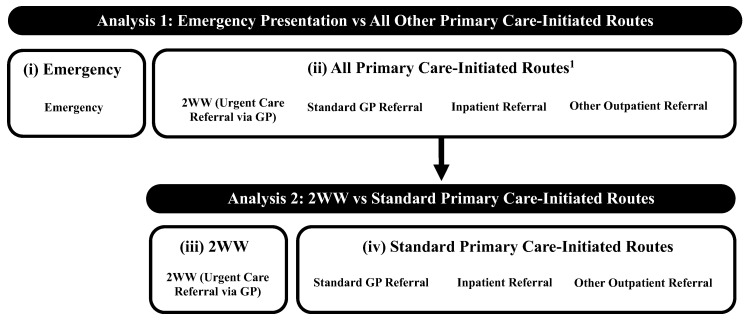
Route to diagnosis categorisation through each analysis. **^1^** These (sub)routes to diagnosis in secondary care all started in primary care; referral may not necessarily have been for suspected lung cancer despite the ‘end point’ of a final lung cancer diagnosis. Categories (i)–(iv) refer to the route to diagnosis sub-categories used in the analysis (see Section 2.3). Abbreviations: GP: General practitioner; 2WW: Two-week wait.

We followed the method of Deane et al. [28] to sub-categorise the RTD for analysis as follows: (i) emergency presentation (comprising (sub)routes: accident and emergency, emergency GP referral, emergency transfer, emergency admission, or attendance); (ii) all primary care-initiated routes (that is, all routes which would have been initiated in primary care as per Elliss-Brookes et al.’s (2012) definition [25]: standard GP, inpatient, or other outpatient referral, or 2WW (urgent care referral from primary care, in this instance from GPs)); (iii) 2WW (urgent care referral from primary care, in this instance via GPs); and (iv) standard primary care-initiated routes (that is, all non-urgent, non-emergency, cancer referral routes: standard GP, inpatient, or other outpatient referral) (Figure 2).

### 2.4. Explanatory Variables

Socio-demographic explanatory variables of interest were categorised as follows: age at diagnosis (<50, 50–59, 60–69, 70–79, 80–80, and 90+); sex (male, female); deprivation category of area of residence (quintile of IMD income domain; quintile 1 includes residents in the least deprived areas and quintile 5 includes residents in the most deprived areas) [29]; ethnicity (White, other ethnic group (Asian/British, Asian, Black/African/Caribbean/Black British, mixed/multiple ethnic groups, and other ethnic groups), or unknown (missing and unknown ethnicity classifications)); rural/urban residence (rural village, hamlet, and isolated dwellings; rural town and fringe; urban city and town; and extensive urban area) based on the Rural Urban Classification for Small Area Geographies in England [30]; and government region (North West, North East, West Midlands, Yorkshire and the Humber, East Midlands, East of England, South East, South West, and London) [31]. Cases with missing or unknown ethnicity information were retained in the dataset as this information may not be missing at random and exclusion could result in bias.

Clinical variables may lie on the pathway between socio-demographic characteristics and the RTD and hence mediate the relationship (e.g., comorbidities) or result from the RTD (e.g., stage). Therefore, these variables were not considered as potential confounders but, instead, have been reported to provide descriptive information about how these variables were distributed across the study population and by RTD. Stage was grouped as TNM I, II, III, IV, or unknown (unknown/missing). Multiple tumours was categorised as “no” (the patient had only the index lung cancer) or “yes” (the patient had the index lung cancer and a prior invasive cancer at another anatomical site). Following SEER classifications and clinical advice [32], tumours were categorised as follows: non-small-cell lung cancer (NSCLC), including adenocarcinoma, squamous cell tumours, large cell tumours, and “not otherwise specified” tumours, small-cell lung cancer (SCLC), and other. Discussion at MDT was grouped as follows: yes, no, or missing. Number of comorbidities was classified as none, 1 to 2, or 3+ and based on the Charlson Comorbidity Index [33]. This index was derived by NCRD colleagues from the number of inpatient hospital admissions between 78 and 6 months pre-diagnosis (disregarding the index lung cancer).

### 2.5. Statistical Analyses

Two separate analyses were conducted. Analysis 1 considered the entire analytical cohort and investigated socio-demographic variations in patients diagnosed through emergency presentation versus all other primary care-initiated routes (i.e., groups (i) and (ii) above). Analysis 2 included only patients diagnosed via the two primary care-initiated routes (category (ii) above) and compared 2WW referral versus standard primary care-initiated routes (i.e., (iii) vs. (iv) above) (Figure 1 and Figure 2).

Baseline descriptive statistics were computed for each analysis. Univariable and, subsequently, multivariable logistic regression models were used to determine the likelihood of diagnosis route by socio-demographic characteristics. Initially, each socio-demographic variable was fitted individually. Then—because our focus was on investigating how different socio-demographic factors are related to RTD—the six socio-demographic variables were fitted simultaneously in multivariable models. We then checked the goodness-of-fit of the two multivariable models; region was dropped from the 2WW vs. standard primary care-initiated route model as it caused poor fit. The final models had adequate fit on the Hosmer–Lemeshow test and mean variance inflation factors of <10. Odds ratios (ORs) and 95% confidence intervals (CIs) have been reported. Throughout, *p* ≤ 0.05 (two-sided) was considered statistically significant. Analyses were conducted in STATA version 16.1 (StataCorp, College Station, TX, USA).

### 2.6. Ethics and Reporting

The study obtained favorable ethical approval from the Proportionate Review Sub-committee of the West Midlands–Edgbaston Research Ethics Committee on 16 October 2019 (REF19/WM/0317). It was performed in accordance with the Declaration of Helsinki and is reported according to the Strengthening the Reporting of Observational Studies in Epidemiology guidelines [34]. Section 251 of the NHS Act 2006 grants legal permission to collect cancer registration information without the need to seek consent. The privacy rights of human subjects were observed.

## 3. Results

### 3.1. Patient Characteristics

A total of 181,763 patients diagnosed with primary lung cancer were included in the study (Table 1). The number of diagnoses was stable over time. Most patients were aged 60–89 (84.2%), of White ethnicity (92.9%), resided in urban areas (82.6%), and had NSCLC histology (86.5%). Almost half were diagnosed at stage IV (48.6%). Diagnosis was associated with residence in areas of higher deprivation (25.8% and 13.9% of patients were residents in the most and least deprived quintiles, respectively). Patient characteristics for each RTD are provided in Supplementary Table S2.

### 3.2. Analysis 1: Emergency Presentation vs. All Primary Care-Initiated Routes

Approximately 35.2% of patients (*n* = 64,045) were diagnosed through emergency presentation, and 64.8% were diagnosed through all primary care-initiated routes (*n* = 117,718) (Table 2). Emergency diagnosis was more frequent among older patients (80–89, 45.2%; 90+, 62.6%) and was slightly more common in females than males (35.8%; 34.8%); those of non-White ethnicities (36.8%); those from more deprived areas (IMD5, 36.9%); and those resident in extensive urban areas (36.8%).

Distributions of emergency diagnoses over time and by clinical variables are shown in Supplementary Table S2. The number of emergency diagnosis declined slightly over time and was more common in those with advanced disease and more comorbidities.

Unadjusted odds ratios for the socio-demographic variables are shown in Supplementary Table S3. When fitted simultaneously in a multivariable model, all socio-demographic variables made a statistically significant contribution (LRT *p* ≤ 0.010). Likelihood of emergency presentation increased as deprivation increased; patients resident in the most deprived areas were 29% more likely to be diagnosed via the emergency route (IMD5 vs. IMD1; multivariable odds ratio (mvOR) 1.29; 95% CI 1.25–1.34) (Table 3). Females were more likely to present as emergencies (female vs. males, mvOR 1.03; 95% CI 1.01–1.05), as were older patients (90+ vs. 70–79 years old, mvOR 3.49; 95% CI 3.32–3.67). Patients resident in rural areas were less likely to present as emergency cases (rural village vs. extensive urban area, mvOR 0.90; 95% CI 0.86–0.94). Geographically, the highest likelihood of emergency presentation was found in patients resident in London (London vs. North West, mvOR 1.27, 95% CI 1.22–1.32).

### 3.3. Analysis 2: 2WW vs. Standard Primary Care-Initiated Routes

In total, 43.7% (51,399) of lung cancer diagnoses initiated in primary care were considered urgent (2WW) compared to 56.3% (66,319) who had standard referral. Urgent referrals were more common in those aged 50–79 (50–59, 45.2%; 60–69, 45.5%; 70–79, 43.1%) and of White ethnicity (44.1%) (Table 2).

Supplementary Table S2 shows the distribution of lung cancers diagnosed through the 2WW pathway by year and clinical variables. There was a small decrease in 2WW pathway diagnosis over time; they were more common in those with later stage disease, no comorbidities, and SCLC histology.

Supplementary Table S3 shows the unadjusted odds ratios for the socio-demographic variables. In the multivariable model including socio-demographic variables, there was a modest increased likelihood of diagnosis through 2WW with increasing deprivation (IMD5 vs. IMD1, mvOR 1.13; 95% CI 1.08–1.17) (Table 3). Likelihood of referral through the 2WW pathway did not vary by sex. Both younger (<50) and older (90+) extremes of age showed a slight decreased likelihood of 2WW diagnosis. Patients with non-White ethnicities were less likely than White patients to be diagnosed through the 2WW (other vs. White, mvOR 0.74; 95% CI 0.70–0.80). Likelihood of diagnosis through the 2WW pathway was one-quarter to one-third more likely in people resident anywhere other than an extensive urban area.

## 4. Discussion

Initiatives to address cancer waiting times in England over the past two decades, in particular the introduction of the 2WW pathway, were intended to standardise and shorten the time to cancer diagnosis. While this is a laudable aim, this population-based analysis of routes to diagnosis in lung cancer in England points to significant socio-demographic inequalities in diagnosis routes (both in emergency presentation and diagnosis through the urgent (2WW) pathway) among those diagnosed with lung cancer. Diagnosis as an emergency (vs. primary care-initiated routes) was associated with increasing deprivation, being female, older, and of other (non-White) ethnicity, and residence in the London region. Diagnosis through the 2WW pathway (versus standard GP referral) was associated with increasing deprivation, being resident outside of an extensive urban area, aged 50–69 years old, and of White ethnicity. Taken together, these findings highlight that emergency and 2WW pathways experience both socio-demographic differences in presentation (e.g., by age and ethnicity) and similarities (e.g., by deprivation status). Given recent changes to cancer waiting time standards, the finding that the route to diagnosis varies by socio-demographics is concerning for optimising referral pathways for all patients.

Socio-demographic associations with emergency presentation are consistent with previous observations that older age and low SES status are linked with lung cancer diagnosis in emergency departments [22,35]. Whilst the causes are likely multifactorial, the ability to recognise and not dismiss symptoms, decide to seek help, navigate the healthcare system, communicate effectively with health professionals, and not view care outcomes as nihilistic are all likely to be important to avoid diagnosis via an emergency route. It was noteworthy that, in this study, the likelihood of diagnosis through an emergency route increased with increasing deprivation in area of residence. Here, deprivation was considered a measure of SES, and there is extensive evidence of an association between higher SES and better access to care [36]. In terms of possible explanations, low SES is associated with suboptimal health literacy, that is, a person’s ability to source, understand, and act on health information [37]. Work in other healthcare settings, not focused on cancer, has reported an association between limited health literacy and greater use of emergency departments [38]. In terms of cancer, UK research has documented a relationship between having lower health literacy and avoiding doctors’ visits and having more fatalistic attitudes towards cancer [39]. This suggests that, in people with lung cancer, suboptimal health literacy may act as a barrier to timely presentation and referral before the point of becoming an emergency; this may—at least in part—explain the observed association.

In terms of other variables, the association between female sex and increased likelihood of being diagnosed as an emergency may reflect one or more of the following: (i) more women are non-smokers [40] and may not consider themselves to be at risk of lung cancer; (ii) women often have increased caring responsibilities and these may form barriers to scheduling and attending appointments; and (iii) women may be considered lower risk by professionals, which could lead to symptoms like cough and breathlessness being overlooked for urgent referral [41].

The observed lower likelihood of emergency presentation among patients resident in rural areas is intriguing, as it contrasts with past findings in other settings regarding the use of emergency departments [42]. While explanations are not obvious, it is important to remember that this study includes only patients diagnosed with cancer. Data (albeit not from England) indicate that lung cancer incidence is lower in individuals living in more rural areas [43], and emergency presentation patterns may, in part, reflect this. Going beyond this, some recent UK work on cancer symptom help-seeking highlights the importance of, and value placed on, continuity of care and strong relationships with local GPs among people in rural communities [44]. This, and the impact that lung cancer symptoms may have on the ability to work, especially in occupations more prevalent in rural areas (e.g., farming), may stimulate rural patients to consult GPs about symptoms, thereby leading to them being less likely to present as emergencies [45]. 

The observed socio-demographic patterning in diagnosis through the urgent vs. standard referral pathway suggests the possibility of health professionals making decisions on referral according to whether the patient is a “typical” lung cancer presentation (i.e., 60–70 years old, smoker, with cough, and with few comorbidities to preclude other working diagnoses). If certain patient groups are less likely to be diagnosed through the 2WW pathway (e.g., extremes of age), this means that alternative diagnostic routes, or strategies, are needed to ensure that no one falls through system “gaps”. Screening may provide a means to “mop up” some patients. However, current guidelines recommend lung checks be offered only to certain groups; those from other groups (e.g., never-smokers and people not meeting the threshold for low-dose CT referral) are not invited for screening [5]. While the Targeted Lung Health Check Programme pilot has been mindful of minimising barriers to screening by demographic characteristics (e.g., resident in specific areas) [6], national screening programme roll-out may need to be supported by initiatives to ensure that this does not become another example of an intervention generated inequality—i.e., the generation of unintended variations in outcomes for sub-groups that arise from a health related intervention [46].

The associations between increasing deprivation level and diagnosis through 2WW might not have been expected a priori given socio-economic inequalities in lung cancer. However, the finding may simply reflect the higher lung cancer incidence among people resident in more deprived areas. On the other hand, smoking prevalence is higher in low socio-economic groups [40]; as a widely recognised risk factor for lung cancer, a patient being a smoker may prompt GPs to make a 2WW (rather than standard) referral. Health literacy may also be relevant here. It is plausible that those patients who present at the GP having recognised symptoms as being potentially concerning and are able to communicate their concerns effectively and advocate for further investigation—skills which are inherent in good health literacy—would be more likely to be referred through the 2WW pathway rather than the standard pathway.

The large population-based nature of this study is a strength. Despite this, the study has several limitations. We did not have access to information on patients who went through these pathways and were not found to have lung cancer; such data would have aided interpretation. In terms of the data we did have access to, as with other registries, data fields on the NCRD are not always complete. For example, a percentage of cases had an unknown RTD (or were coded as DCO) and were excluded from analysis. These excluded cases were somewhat older, less deprived, and less often of White ethnicity than the included cases. While it was out of the scope of the current study to impute an unknown RTD, this may be of value in future research to help minimise bias. In addition, ethnicity data are poorly recorded in NHS records (as borne out here), which limits our ability to comment on different ethnic groups. We did not have any reason to assume a linear relationship between age and outcomes, so we chose to categorise age; however, this may not fully capture the functional form of the relationships. In addition, factors that may be implicated in how a patient presents for diagnosis (e.g., smoking status) were not available; thus, associations between all of the variables that are associated with smoking prevalence (e.g., deprivation and sex) and RTD are likely to be affected by uncontrolled confounding. Regarding analysis, we chose to conduct two separate (albeit complementary) analyses to address our research questions with the intention of providing results that would be readily interpretable by decision-makers and service providers. A multinomial regression with a three-level outcome variable (emergency/2WW/standard GP referral) would have reduced the number of statistical tests conducted and the possibility of associations being generated by chance. Finally, these analyses are obviously specific to English referral pathways, which limits their wider generalisability.

There are multiple policy, practice, and research implications of this work. From a research perspective, the persistence of emergency presentation as the most common route of diagnosis, even when compared to other countries internationally, is concerning [1,47]. These analyses suggest that there has been only a small reduction in the percentage of patients presenting via emergencies (from 39% to 35%, respectively) and a small increase in the number of 2WW referrals (from 24% to 28%, respectively) since Elliss-Brookes et al.’s 2006–2008 analysis of English RTD data [25]. Further research is needed to better understand the reasons why, with considerable focus in the NHS in England on earlier cancer diagnosis, these figures are relatively unchanged; qualitative studies in this context may be illuminating. In addition, an updated analysis covering more recent years, including the pandemic time period, is warranted, given that COVID-19 measures (e.g., social distancing, self-isolation, and the number of patients seen at any one time) are known to have impacted urgent cancer diagnostic pathways [48]. In terms of policy and practice, whilst screening will provide a new diagnostic pathway for lung cancer, GPs will likely remain gatekeepers to diagnosis for patients coming through the symptomatic pathway. Hence, optimisation of referral pathways from GPs remains a priority. One potential solution could be a respiratory system “helpline” for X-ray referral to increase access to cancer diagnostics in secondary/tertiary care for all patients. Alternative approaches that enable patients to bypass their GP and directly access investigations could also be of value. For example, some walk-in urgent treatment centres in the community in England already have facilities for taking X-rays; the extension of this service, supported by appropriate expertise to interpret images, may be worth considering. Additionally, raising awareness of lung cancer symptoms, increasing education to reduce stereotyping (i.e., lung cancer is not just a disease that occurs among smokers and men), and minimising the stigma of diagnosis [4] may help prompt help-seeking from wider demographic groups. Finally, it is imperative that RTD monitoring extends beyond target setting for the new 28 days Faster Diagnosis Standard to also include patterning by socio-demographics—with early action being taken if inequalities are observed.

## 5. Conclusions

This population-based analysis shows that socio-demographic variation is present in lung cancer routes to diagnosis in England—including both emergency presentation and urgent cancer referrals. Whilst screening implementation is an important step towards earlier diagnosis, careful monitoring of this and the new cancer referral targets must be undertaken to ensure that inequalities in diagnosis do not widen.

## Figures and Tables

**Table 1:** Demographic and clinical characteristics of the cohort with a first invasive primary lung cancer stage I–IV/unknown diagnosed between 1 January 2012 and 31 December 2016 (*n* = 181,763).

Characteristic	Number (%)
**Deprivation ^1^**	
1 (Least Deprived)	25,302 (13.92)
2	32,606 (17.94)
3	36,234 (19.93)
4	40,685 (22.38)
5 (Most Deprived)	46,936 (25.82)
**Sex**	
Male	97,827 (53.82)
Female	83,936 (46.18)
**Age at Diagnosis (Years)**	
<50	4632 (2.55)
50–59	16,772 (9.23)
60–69	47,667 (26.22)
70–79	63,050 (34.69)
80–89	42,325 (23.29)
90+	7317 (4.03)
**Ethnicity**	
White	168,809 (92.87)
Other Ethnic Group ^2^	6256 (3.44)
Unknown ^3^	6698 (3.69)
**Rural/Urban Residence**	
Rural Village, Hamlet, and Isolated Dwellings	14,231 (7.83)
Rural Town and Fringe	17,439 (9.59)
Urban City and Town	80,331 (44.20)
Extensive Urban Area	69,762 (38.38)
**Government Region**	
North West	30,903 (17.00)
North East	13,301 (7.32)
West Midlands	18,661 (10.27)
Yorkshire and the Humber	21,535 (11.85)
East Midlands	15,654 (8.61)
East of England	18,765 (10.32)
South East	26,027 (14.32)
South West	17,738 (9.76)
London	19,179 (10.55)
**Stage at Diagnosis**	
I	27,476 (15.12)
II	13,520 (7.44)
III	35,303 (19.42)
IV	88,363 (48.61)
Unknown ^4^	17,101 (9.41)
**Histology**	
SCLC	19,125 (10.52)
NSCLC ^5^	157,214 (86.49)
Other ^6^	5424 (2.98)
**Multiple Tumours ^7^**	
No	149,148 (82.06)
Yes	32,615 (17.94)
**Number of Comorbidities ^8^**	
0	101,797 (56.01)
1–2	56,059 (30.84)
3+	23,907 (13.15)
**Discussed at MDT**	
Yes	83,489 (45.93)
No	30,631 (16.85)
Missing	67,643 (37.21)
**Diagnosis Year**	
2012	36,067 (19.84)
2013	36,157 (19.89)
2014	36,506 (20.08)
2015	36,516 (20.09)
2016	36,517 (20.09)
**Diagnosis Route ^9^**	
Emergency ^10^	64,045 (35.24)
Standard GP Referral	41,788 (22.99)
Inpatient Elective	2976 (1.64)
Outpatient (Other)	21,555 (11.86)
2WW ^11^	51,399 (28.28)
All Primary Care-Initiated Routes ^12^	117,718 (64.76)
Standard Primary Care-Initiated Routes ^13^	66,319 (36.49)

^1^ Refers to IMD (income domain). For diagnosis year 2012, IMD_2010 was used, and for diagnosis years 2013–2016, IMD_2015 was used. ^2^ Other ethnic group refers to Asian/British Asian, Black/African/Caribbean/Black British, mixed/multiple ethnic groups, and other ethnic groups. ^3^ Unknown ethnicity refers to unknown and missing ethnicity classifications. ^4^ Unknown staging refers to missing and unstageable tumours. ^5^ NSCLC refers to adenocarcinomas, squamous cell, large cell, and not otherwise specified NSCLC. ^6^ Other histology refers to other specified and non-specified lung cancers. ^7^ Refers to any invasive tumour(s) other than the index lung cancer. Yes, indicates that the patient had previously (i.e., before the current lung cancer diagnosis) been diagnosed with another cancer. ^8^ Refers to number of comorbidities between 78 and 6 months prior to diagnosis as determined using the Charlson Comorbidity Index. ^9^ Reported as a percentage of the analytical population (*n* = 181,763). ^10^ Emergency refers to A&E, emergency GP referral, emergency transfer, and emergency admission or attendance. ^11^ 2WW refers to GP referral via the Urgent Care Pathway. ^12^ All primary care-initiated routes refers to standard GP referral, inpatient referral, outpatient (other), and 2WW (urgent care referral via GP). ^13^ Standard Primary Care-Initiated Routes refers to standard GP referral, inpatient referral, and other outpatient referral. Abbreviations: IMD: Index of Multiple Deprivation (income domain); MDT: Multi-disciplinary team; NSCLC: Non-small-cell lung cancer; SCLC: Small-cell lung cancer; 2WW: Two-week wait.

**Table 2:** Routes to diagnosis for patients with lung cancer diagnosed between 1 January 2012 and 31 December 2016, analyses 1 and 2, numbers and percentages.

	Analysis 1: Emergency Presentation vs. All Primary Care-Initiated Routes		Analysis 2: 2WW vs. Standard Primary Care-Initiated Routes	
	Emergency Presentation ^1^ *n* = 64,045 (35.24%)	All Primary Care-Initiated Routes ^2^ *n* = 117,718 (64.76%)	2WW ^3^ *n* = 51,399 (43.66%)	Standard Primary Care-Initiated Routes ^4^ *n* = 66,319 (56.34%)
**Deprivation ^4,5^**				
1 (Least Deprived)	8193 (32.38)	17,109 (67.62)	7234 (42.28)	9875 (57.72)
2	10,976 (33.66)	21,630 (66.34)	9569 (44.24)	12,061 (55.76)
3	12,751 (35.19)	23,483 (64.81)	10,300 (43.86)	13,183 (56.14)
4	14,816 (36.42)	25,869 (63.58)	11,476 (44.36)	14,393 (55.64)
5 (Most Deprived)	17,309 (36.88)	29,627 (63.12)	12,820 (43.27)	16,807 (56.73)
**Sex**				
Male	34,022 (34.78)	63,805 (65.22)	27,805 (43.58)	36,000 (56.42)
Female	30,023 (35.77)	53,913 (64.23)	23,594 (43.76)	30,319 (56.24)
**Age at Diagnosis (Years)**				
<50	1471 (31.76)	3161 (68.24)	1276 (40.37)	1885 (59.63)
50–59	4896 (29.19)	11,876 (70.81)	5367 (45.19)	6509 (54.81)
60–69	13,466 (28.25)	34,201 (71.75)	15,574 (45.54)	18,627 (54.46)
70–79	20,487 (32.49)	42,563 (67.51)	18,333 (43.07)	24,230 (56.93)
80–89	19,147 (45.24)	23,178 (54.76)	9708 (41.88)	13,470 (58.12)
90+	4578 (62.57)	2739 (37.43)	1141 (41.66)	1598 (58.34)
**Ethnicity**				
White	58,809 (34.84)	110,000 (65.16)	48,528 (44.12)	61,472 (55.88)
Other Ethnic Group ^6^	2300 (36.76)	3956 (63.24)	1396 (35.29)	2560 (64.71)
Unknown ^7^	2936 (43.83)	3762 (56.17)	1475 (39.21)	2287 (60.79)
**Rural/Urban Residence**				
Rural Village, Hamlet, and Isolated Dwellings	4478 (31.47)	9753 (68.53)	4361 (44.71)	5392 (55.29)
Rural Town and Fringe	5920 (33.95)	11,519 (66.05)	5248 (45.56)	6271 (54.44)
Urban City and Town	27,978 (34.83)	52,353 (65.17)	24,321 (46.46)	28,032 (53.54)
Extensive Urban Area	25,669 (36.80)	44,093 (63.20)	17,469 (39.62)	26,624 (60.38)
**Government Region ^8^**				
North West	10,395 (33.64)	20,508 (66.36)	-	-
North East	4640 (34.88)	8661 (65.12)	-	-
West Midlands	6642 (35.59)	12,019 (64.41)	-	-
Yorkshire and the Humber	7803 (36.23)	13,732 (63.77)	-	-
East Midlands	5559 (35.51)	10,095 (64.49)	-	-
East of England	6386 (34.03)	12,379 (65.97)	-	-
South East	8892 (34.16)	17,135 (65.84)	-	-
South West	6091 (34.34)	11,647 (65.66)	-	-
London	7637 (39.82)	11,542 (60.18)	-	-

^1^ Emergency refers to A&E, emergency GP referral, emergency transfer, and emergency admission or attendance. ^2^ All primary care-initiated routes refers to standard GP referral, inpatient referral, outpatient (other) referral, and 2WW (urgent care referral via GP). ^3^ 2WW refers to GP referral via the urgent cancer pathway. ^4^ Standard Primary Care-Initiated Routes refers to standard GP referral, inpatient referral, and other outpatient referral. ^5^ Refers to IMD (income domain). For diagnosis year 2012, IMD_2010 was used, and for diagnosis years 2013–2016, IMD_2015 was used. ^6^ Other ethnic group refers to Asian/British Asian, Black/African/Caribbean/Black British, mixed/multiple ethnic groups, and other ethnic groups. ^7^ Unknown ethnicity refers to missing and unknown ethnicity classifications. ^8^ Not shown for analysis 2 as the variable was excluded from the multivariable model. Abbreviations: IMD: Index of Multiple Deprivation; 2WW: Two week wait.

**Table 3:** Multivariable analyses of associations between socio-demographic variables and the route to diagnosis for patients with lung cancer diagnosed between 1 January 2012 and 31 December 2016: Likelihood of (i) emergency presentation (diagnosis via the emergency route versus all primary care-initiated routes, *n* = 181,763) and (ii) referral urgency (diagnosis via the 2WW pathway (urgent referral via GP) vs. all other standard primary care-initiated routes, *n* = 117,718). Multivariable odds ratios (mvOR) with 95% confidence intervals.

	Analysis 1: Emergency Presentation vs. All Primary Care-Initiated Routes ^1^		Analysis 2: 2WW Pathway vs. All Other Standard Primary Care-Initiated Routes ^2^	
	mvOR	95% CI	mvOR	95% CI
**Deprivation ^3^**				
1 (Least Deprived)	1.00	-	1.00	-
2	1.07	1.03–1.11	1.08	1.04–1.13
3	1.15	1.11–1.19	1.08	1.04–1.12
4	1.22	1.18–1.27	1.13	1.08–1.17
5 (Most Deprived)	1.29	1.25–1.34	1.13	1.08–1.17
**Sex**				
Male	1.00	-	1.00	-
Female	1.03	1.01– 1.05	1.00	0.98–1.03
**Age at Diagnosis (Years)**				
<50	0.93	0.87–0.99	0.92	0.85–0.99
50–59	0.83	0.80–0.87	1.10	1.05–1.14
60–69	0.81	0.79–0.83	1.10	1.07–1.13
70–79	1.00	-	1.00	-
80–89	1.73	1.68–1.77	0.95	0.92–0.98
90+	3.49	3.32–3.67	0.95	0.87–1.02
**Ethnicity**				
White	1.00	-	1.00	-
Other Ethnic Group ^4^	1.05	0.99–1.11	0.74	0.70–0.80
Unknown ^5^	1.40	1.33–1.48	0.81	0.75–0.86
**Rural/Urban Residence**				
Rural Village, Hamlet, and Isolated Dwellings	0.90	0.86–0.94	1.27	1.21–1.33
Rural Town and Fringe	0.94	0.91–0.98	1.29	1.24–1.35
Urban City and Town	0.97	0.95–1.00	1.33	1.29–1.36
Extensive Urban Area	1.00	-	1.00	-
**Government Region**				
North West	1.00	-	-	-
North East	1.04	1.00–1.09	-	-
West Midlands	1.10	1.05–1.14	-	-
Yorkshire and the Humber	1.13	1.09–1.17	-	-
East Midlands	1.13	1.08–1.18	-	-
East of England	1.06	1.02–1.10	-	-
South East	1.06	1.02–1.10	-	-
South West	1.07	1.03–1.12	-	-
London	1.27	1.22–1.32	-	-

^1^ Model 1—emergency presentations vs. all primary care-initiated routes; variables mutually adjusted; all variables LRT *p* ≤ 0.010; Hosmer–Lemeshow chi-square (8df) = 9.31, *p* = 0.371; variance inflation factor for variables in model, mean = 1.05, range 1.00–1.15. Emergency refers to A&E, emergency GP referral, emergency transfer, and emergency admission or attendance. All primary care-initiated routes refers to standard GP referral, inpatient referral, outpatient (other) referral, and 2WW (urgent care referral via GP). ^2^ Model 2—diagnosis via the 2WW pathway vs. all other standard primary care-initiated routes; variables shown have been mutually adjusted; government region dropped due to poor fit; all other variables (with exception of sex) LRT *p* < 0.001; LRT sex *p* = 0.768; Hosmer–Lemeshow chi-square (8df) = 5.94, *p* = 0.654; variance inflation factor for variables in model, mean = 1.05, range 1.00–1.13. 2WW refers to GP referral via the urgent cancer pathway. ^3^ Refers to IMD (income domain). For diagnosis year 2012, IMD_2010 was used, and for diagnosis years 2013–2016, IMD_2015 was used. ^4^ Other ethnic group refers to Asian/British Asian, Black/African/Caribbean/Black British, mixed/multiple ethnic groups, and other ethnic groups. ^5^ Unknown ethnicity refers to missing and unknown ethnicity classifications. Abbreviations: IMD: Index of Multiple Deprivation; mvOR: multivariable odds ratio; 95% CI: 95% confidence interval; 2WW: Two-week wait.

## Data Availability

The data used in this analysis were released to the authors for the purposes of this work, and they have permission to share them. Applications for these data can be made with the current Data Controller—NHS England for anyone wishing to access this information.

## Supplementary Materials

The following supporting information can be downloaded at: https://www.mdpi.com/article/10.3390/cancers17111874/s1. Table S1. Demographic and clinical characteristics of the invasive primary lung cancers diagnosed between 1 January 2012–31 December 2016 which were excluded from analysis on basis of unknown RTD or being DCO (*n* = 5938). Table S2. Demographic and clinical characteristics of all patients with a first primary invasive lung tumour stage I-IV/unknown diagnosed 2012–2016 by diagnosis route (*n* = 181,763). Table S3. Unadjusted analyses of associations between socio-demographic variables and route to diagnosis for patients with lung cancer diagnosed between 1 February 2012-31 December 2016: Likelihood of (i) emergency presentation (diagnosis via the emergency route versus all primary care-initiated routes, *n* = 181,763) and (ii) referral urgency (diagnosis via the 2WW pathway (urgent referral via GP) vs all other standard primary care-initiated routes, *n* = 117,718). Unadjusted odds ratios (OR) with 95% confidence intervals.

## Abbreviations

CI: Confidence interval; DCO: Death certificate only; GP: General practitioner; ICD: International Classification of Diseases; IMD: Index of multiple deprivation; LRT: Likelihood ratio test; MDT: Multidisciplinary team; mvOR: Multivariable odds ratio; NCRD: National Cancer Registry Database; NHS: National Health Service; NSCLC: Non-small-cell lung cancer; OR: Odds ratio; RTD: Route(s) to diagnosis; SCLC: Small-cell lung cancer; SEER: Surveillance, Epidemiology, and End Results Program; SES: Socio-economic status; TNM: Tumour, Nodes, Metastases; UK: United Kingdom; 2WW: Two-week wait.

## References

[1] The United Kingdom Lung Cancer Coalition (2020). Early Diagnosis Matters: Making the Case for the Early and Rapid Diagnosis of Lung Cancer. https://www.uklcc.org.uk/our-reports/january-2020/early-diagnosis-matters.

[2] McPhail S., Johnson S., Greenberg D., Peake M., Rous B. (2015). Stage at diagnosis and early mortality from cancer in England. Br. J. Cancer.

[3] Arnold M., Rutherford M., Bardot A., Ferlay J., Andersson T.M.-L., Myklebust T.Å., Tervonen H., Thursfield V., Ransom D., Shack L. (2019). Progress in cancer survival, mortality, and incidence in seven high-income countries 1995-2014 (ICBP SURVMARK-2): A population-based study. Lancet Oncol..

[4] Sanghrajka A., Sharp L., Rowlands G. How does socio-economic disadvantage influence the timeliness of lung cancer diagnosis? A systematic review and synthesis of published qualitative studies. Pub. Health.

[5] Department of Health & Social Care (2023). New Lung Cancer Screening Roll out to Detect Cancer Sooner. https://www.gov.uk/government/news/new-lung-cancer-screening-roll-out-to-detect-cancer-sooner.

[6] Crosbie P.A., Balata H., Evison M., Atack M., Bayliss-Brideaux V., Colligan D., Duerden R., Eaglesfield J., Edwards T., Elton P. (2019). Implementing lung cancer screening: Baseline results from a community-based “Lung Health Check” pilot in deprived areas of Manchester. Thorax.

[7] Horst C., Dickson J.L., Tisi S., Ruparel M., Nair A., Devaraj A., Janes S.M. (2020). Delivering low-dose CT screening for lung cancer: A pragmatic approach. Thorax.

[8] Department of Health (2000). The NHS Cancer Plan: A Plan for Investment, a Plan for Reform. https://image.guardian.co.uk/sys-files/Society/documents/2003/08/26/cancerplan.pdf.

[9] Round T., Ashworth M., L’esperance V., Møller H. (2021). Cancer detection via primary care urgent referral and association with practice characteristics: A retrospective cross-sectional study in England from 2009/2010 to 2018/2019. Br. J. Gen. Pract..

[10] Neal R.D., Smith L. (2021). Urgent cancer referrals: How well are they working and can they be improved?. Br. J. Gen. Pract..

[11] National Cancer Institute (2023). Suspected Cancer: Recognition and Referral NICE Guideline NG12. https://www.nice.org.uk/guidance/ng12.

[12] NHS England (2023). Implementing a Timed Lung Cancer Diagnostic Pathway. https://www.england.nhs.uk/long-read/implementing-a-timed-lung-cancer-diagnostic-pathway/.

[13] Onwuka J.U., Zahed H., Feng X., Alcala K., Erhunmwunsee L., Williams R.M., Aldrich M.C., Ahluwalia J.S., Albanes D., Arslan A.A. (2025). Association between socioeconomic position and lung cancer incidence in 16 countries: A prospective cohort consortium study. eClinicalMedicine.

[14] Martins T., Abel G., Ukoumunne O.C., Price S., Lyratzopoulos G., Chinegwundoh F., Hamilton W. (2022). Assessing ethnic inequalities in diagnostic interval of common cancers: A population-based UK cohort study. Cancers.

[15] Norris R.P., Dew R., Greystoke A., Todd A., Sharp L. (2023). Socio-economic inequalities in novel NSCLC treatments during the era of tumor biomarker guided therapy: A population-based cohort study in a publicly funded healthcare system. J. Thorac. Oncol..

[16] Martins T., Abel G., Okumunne O.C., Mounce L.T.A., Price S., Lyratzopoulos G., Hamilton W. (2022). Ethnic inequalities in routes to diagnosis of cancer: A population-based UK cohort study. Br. J. Cancer.

[17] O’Dowd E.L., McKeever T.M., Baldwin D.R., Anwar S., Powell H.A., Gibson J.E., Iyen-Omofoman B., Hubbard R.B. (2015). What characteristics of primary care and patients are associated with early death in patients with lung cancer in the UK?. Thorax.

[18] Beard E., Brown J., Jackson S.E., West R., Kock L., Boniface S., Shahab L. (2021). Independent Associations Between Different Measures of Socioeconomic Position and Smoking Status: A Cross-Sectional Study of Adults in England. Nicotine Tob. Res..

[19] Lei F., Lee E. (2019). Barriers to lung cancer screening with low-dose computed tomography. Oncol. Nurs. Forum..

[20] Carter-Harris L., Brandzel S., Wernli K.J., Roth J.A., Buist D.S.M. (2017). A qualitative study exploring why individuals opt out of lung cancer screening. Fam. Pract..

[21] Danckert B., Christensen N.L., Falborg A.Z., Frederiksen H., Lyratzopoulos G., McPhail S., Pederson A.F., Ryg J., Thomsen L.A., Vedsted P. (2022). Assessing how routes to diagnosis vary by the age of patients with cancer: A nationwide register-based cohort study in Denmark. BMC Cancer.

[22] Abel G.A., Shelton J., Johnson S., Elliss-Brookes L., Lyratzopoulos G. (2015). Cancer-specific variation in emergency presentation by sex, age and deprivation across 27 common and rarer cancers. Br. J. Cancer.

[23] Danckert B., Falborg A.Z., Christensen N.L., Frederiksen H., Lyratzopoulos G., McPhail S., Ryg J., Vedsted P., Thomsen L.A., Jensen H. (2021). Routes to diagnosis and the association with the prognosis in patients with cancer—A nationwide register-based cohort study in Denmark. Cancer Epidemiol..

[24] Henson K.E., Ellis-Brookes L., Coupland V.H., Payne E., Vernon S., Rous B., Rashbass J. (2020). Data resource profile: National cancer registration dataset in England. Int. J. Epidemiol..

[25] Elliss-Brookes L., McPhail S., Ives A., Greenslade M., Shelton J., Hiom S., Richards M. (2012). Routes to diagnosis for cancer—Determining the patient journey using multiple routine data sets. Br. J. Cancer.

[26] Union for International Cancer Control (2016). TNM: Classification of Malignant Tumours.

[27] Fritz A., Percy C., Jack A., Shanmugaratnam K., Sobin L., Parkin D.M., Whelan S., World Health Organisation (2000). International Classification of Disease for Oncology (ICD-O3).

[28] Deane J., Norris R., O’Hara J., Patterson J., Sharp L. (2022). Who presents where? a population-based analysis of socio-demographic inequalities in head and neck cancer patients’ referral routes. Int. J. Environ. Res. Public Health.

[29] Department for Communities and Local Government (2015). The English Index of Multiple Deprivation (IMD) 2015—Guidance. https://assets.publishing.service.gov.uk/government/uploads/system/uploads/attachment_data/file/464430/English_Index_of_Multiple_Deprivation_2015_-_Guidance.pdf.

[30] Department for Environment, Food & Rural Affairs (2021). Rural Urban Classification. https://www.gov.uk/government/collections/rural-urban-classification#:~:text=The%20six%20categories%20are%3A,population%20resides%20in%20rural%20areas.

[31] Office for National Statistics England: Detailed Information on the Administrative Structure Within England. https://www.ons.gov.uk/methodology/geography/ukgeographies/administrativegeography/england.

[32] Yu M., Feuer E.J., Cronin K.A., Caporaso N.E. (2014). Use of multiple imputation to correct for bias in lung cancer incidence trends by histologic subtype. Cancer Epidemiol. Biomark. Prev..

[33] Charlson M.E., Pompei P., Ales K.L., MacKenzie C.R. (1987). A new method of classifying prognostic comorbidity in longitudinal studies: Development and validation. J. Chronic Dis..

[34] Von Elm E., Altman D.G., Egger M., Pocock S.J., Gøtzsche P.C., Vanderbroucke J.P. (2007). The strengthening the reporting of observational studies in epidemiology (STROBE) statement: Guidelines for reporting observational studies. Ann. Intern. Med..

[35] Beckett P., Tata L.J., Hubbard R.B. (2014). Risk factors and survival outcome for non-elective referral in non-small cell lung cancer patients-analysis based on the national lung cancer audit. Lung Cancer.

[36] McMaughan D.J., Oloruntoba O., Smith M.L. (2020). Socioeconomic Status and Access to Healthcare: Interrelated Drivers for Healthy Aging. Front. Public Health.

[37] Public Health England (2015). Local Action on Health Inequalities Improving Health Literacy to Reduce Health Inequalities. https://assets.publishing.service.gov.uk/government/uploads/system/uploads/attachment_data/file/460710/4b_Health_Literacy-Briefing.pdf.

[38] Schumacher J.R., Hall A.G., Davis T.C., Arnold C.L., Bennett R.D., Wolf M.S., Carden D.L. (2013). Potentially Preventable Use of Emergency Services: The Role of Low Health literacy. Med. Care.

[39] Morris N.S., Field T.S., Wagner J.L., Cutrona S.L., Roblin D.W., Gaglio B., Williams A.E., Han P.J.K., Costanza M.E., Mazor K.M. (2013). The association between health literacy and cancer-related attitudes, behaviors, and knowledge. J. Health Commun..

[40] Office for National Statistics (2022). Adult Smoking Habits in the UK: 2022. https://www.ons.gov.uk/peoplepopulationandcommunity/healthandsocialcare/healthandlifeexpectancies/bulletins/adultsmokinghabitsingreatbritain/2022.

[41] Health Services Safety Investigations Body (2021). Investigations Report: Missed Detection of Lung Cancer on Chest X-Rays of Patients Being Seen in Primary Care. https://www.hssib.org.uk/patient-safety-investigations/missed-detection-of-lung-cancer-on-chest-x-rays-of-patients-being-seen-in-primary-care/investigation-report/.

[42] Greenwood-Ericksen M.B., Kocher K. (2019). Trends in emergency department use by rural and urban populations in the United States. JAMA Netw. Open.

[43] Sharp L., Donnelly D., Hegarty A., Carsin A.-E., Deady S., McCluskey N., Gavin A., Comber H. (2014). Risk of several cancers is higher in urban areas after adjusting for socioeconomic status. Results from a two-country population-based study of 18 common cancers. J. Urban Health.

[44] Dobson C., Deane J., MacDonald S., Murchie P., Ellwood C., Angell L., Rubin G. (2022). Barriers to early presentation amongst rural residents experiencing symptoms of colorectal cancer: A qualitative interview study. Cancers.

[45] Bergin R.J., Emery J., Bollard R.C., Falborg A.Z., Jensen H., Weller D., Menon U., Vedsted P., Thoma R.J., Whitfield K. (2018). Rural–urban disparities in time to diagnosis and treatment for colorectal and breast cancer. Cancer Epidemiol. Biomark. Prev..

[46] Lorenc T., Petticrew M., Welch V., Tugwell P. (2013). What types of interventions generate inequalities? Evidence from systematic reviews. J. Epidemiol. Community Health.

[47] McPhail S., Swabb R., Johnson S.A., Barclay M.E., Elkader H.A., Alvi R., You H. (2022). Risk factors and prognostic implications of diagnosis of cancer within 30 days after an emergency hospital admission (emergency presentation): An international cancer benchmarking partnership (ICBP) population-based study. Lancet Oncol..

[48] NHS England (2021). Urgent Cancer Diagnostic Services During COVID-19. https://www.england.nhs.uk/coronavirus/documents/c0789-urgent-cancer-diagnostic-services-during-covid-19/#:~:text=Urgent%20cancer%20diagnostic%20pathways%20have,be%20seen%20at%20one%20time.

