# Supplementary material for: Routes to Diagnosis in Lung Cancer—Do Socio-Demographics Matter? An English Population-Based Study
Source: Cancers (Basel). 2025 Jun 3;17(11):1874. doi: 10.3390/cancers17111874 (PMC12153770; doi:10.3390/cancers17111874)
Supplement: Supplementary file 1 [file cancers-17-01874-s001.zip › cancers-3539105-supplementary.pdf]

Supplementary Material

Routes to Diagnosis in Lung Cancer— Do Socio-Demographics Matter? An English Population-Based Study

Ruth P. Norris [1], Elizabeth Fuller [2], Alastair Greystoke [3], Adam Todd [4] and Linda Sharp [1,*]

Supplementary Table S1 Demographic and clinical characteristics of the invasive primary lung cancers diagnosed between 01/01/2012 - 31/12/2016 which were excluded from analysis on basis of unknown RTD or being DCO
Supplementary Table S2 Demographic and clinical characteristics by diagnosis route
Supplementary Table S3 Unadjusted analyses of associations between socio-demographic variables and route to diagnosis for patients with lung cancer diagnosed between 01/02/2012-31/12/2016

Supplementary Table S1. Demographic and clinical characteristics of the invasive primary lung cancers diagnosed between 01/01/2012 - 31/12/2016 which were excluded from analysis on basis of unknown RTD or being DCO (*n*=5,938).

Characteristic	Number (%)
**Deprivation**[1]	
1 (Least Deprived)	1,010 (17.01%)
2	1,084 (18.26%)
3	1,162 (19.57%)
4	1,240 (20.88%)
5 (Most Deprived)	1,441 (24.27%)
**Sex**	
Male	3,133 (52.76%)
Female	2,805 (47.24%)
**Age at Diagnosis (Years)**	
<50	157 (2.64%)
50-59	458 (7.71%)
60-69	1,335 (22.48%)
70-79	1,839 (30.97%)
80-89	1,673 (28.17%)
90+	476 (8.02%)
**Ethnicity**	
White	4,114 (69.28%)
Other Ethnic Group[2]	204 (3.44%)
Unknown[3]	1,620 (27.28%)
**Rural/Urban Residence**	
Rural Village, Hamlet & Isolated Dwellings	539 (9.08%)
Rural Town & Fringe	530 (8.93%)
Urban City & Town	2,688 (45.27%)
Extensive Urban Area	2,181 (36.73%)
**Government Region**	
North West	846 (14.25%)
North East	355 (5.98%)
West Midlands	610 (10.27%)
Yorkshire & the Humber	578 (9.73%)
East Midlands	453 (7.63%)
East of England	595 (10.02%)
South East	1,105 (18.61%)
South West	612 (10.31%)
London	784 (13.20%)

Supplementary Table S1. Continued.

Characteristic	Number (%)
**Stage at Diagnosis**	
I	722 (12.16%)
II	283 (4.77%)
III	577 (9.72%)
IV	1,801 (30.33%)
Unknown[4]	2,555 (43.03%)
**Histology**	
SCLC	261 (4.40%)
NSCLC[5]	116 (1.95%)
Other[6]	5,561 (93.65%)
**Multiple Tumors[7]**	
No	4,961 (83.55%)
Yes	977 (16.45%)
**Number of Comorbidities[8]**	
0	3,767 (63.44%)
1-2	1,511 (25.45%)
3+	660 (11.11%)
**Discussed at MDT**	
Yes	1,108 (18.66%)
No	573 (9.65%)
Missing	4,257 (71.69%)
**Diagnosis Year**	
2012	955 (16.08%)
2013	1,093 (18.41%)
2014	1,085 (18.27%)
2015	1,152 (19.40%)
2016	1,653 (27.84%)

[1]Refers to IMD (income domain). For diagnosis year 2012, IMD_2010 was used and for diagnosis years 2013-2016, IMD_2015 was used. 1 case had unknown deprivation category and has been excluded. [2]Other ethnic group refers to Asian/British Asian, Black/African/Caribbean/Black British, mixed/multiple ethnic groups and other ethnic groups. [3]Unknown ethnicity refers to unknown and missing ethnicity classifications. [4]Unknown staging refers to missing and unstageable tumors. [5]NSCLC refers to adenocarcinomas, squamous cell, large cell, and not otherwise specified NSCLC. [6]Other histology refers to other specified and non-specified lung cancers. [7]Refers to any tumor(s) other than the index lung cancer. Yes, indicates that the patient had previously (i.e. before the current lung cancer diagnosis) been diagnosed with another cancer. [8]Refers to number of comorbidities between 78 to 6 months prior to diagnosis as determined by the Charlson Comorbidity Index. Abbreviations: IMD: Index of Multiple Deprivation (income domain); MDT: Multi-disciplinary team; NSCLC: Non-small cell lung cancer; SCLC: Small cell lung cancer.

Supplementary Table S2. Demographic and clinical characteristics of all patients with a first primary invasive lung tumour stage I-IV/unknown diagnosed 2012-2016 by diagnosis route (*n*=181,763).

Analysis 1: Emergency Presentation		Emergency[1]	All Primary Care-Initiated Routes[2]			
**Analysis 2: Referral Urgency**				Standard Primary Care-Initiated Routes[4]		
	**Overall** (n=181,763; 100%)	**Emergency** (n= 64,045; 35.24%)	**2WW[3]** (n=51,399; 28.28%)	**Standard GP Referral** (n=41,788; 22.99%)	**Inpatient Referral** (n= 2,976; 1.64%)	**Other Outpatient Referral** (n=21,555; 11.86%)
**Deprivation[5]**						
1 (Least Deprived)	25,302 (13.92)	8,193 (12.79)	7,234 (14.07)	6,205 (14.85)	493 (16.57)	3,177 (14.74)
2	32,606 (17.94)	10,976 (17.14)	9,569 (18,.62)	7,650 (18.31)	555 (18.65)	3,856 (17.89)
3	36,234 (19.93)	12,751 (19.91)	10,300 (20.04)	8,301 (19.86)	593 (19.93)	4,289 (19.90)
4	40,685 (22.38)	14,816 (23.13)	11,476 (22.33)	9,151 (21.90)	615 (20.67)	4,627 (21.47)
5 (Most Deprived)	46,936 (25.82)	17,309 (27.03)	12,820 (24.94)	10,481 (25.08)	720 (24.19)	5,606 (26.01)
**Sex**						
Male	97,827 (53.82)	34,022 (53.12)	27,805 (54.10)	22,725 (54.38)	1,597 (53.66)	11,678 (54.18)
Female	83,936 (46.18)	30,023 (46.88)	23,594 (45.90)	19,063 (45.62)	1,379 (46.34)	9,877 (45.82)
**Age at Diagnosis (Years)**						
<50	4,632 (2.55)	1,471 (2.30)	1,276 (2.48)	1,059 (2.53)	153 (5.14)	673 (3.12)
50-59	16,772 (9.23)	4,896 (7.64)	5,367 (10.44)	3,787 (9.06)	430 (14.45)	2,292 (10.63)
60-69	47,667 (26.22)	13,466 (21.03)	15,574 (30.30)	11,354 (27.17)	999 (33.57)	6,274 (29.11)
70-79	63,050 (34.69)	20,487 (31.99)	18,333 (35.67)	15,458 (36.99)	968 (32.53)	7,804 (36.21)
80-89	42,325 (23.29)	19,147 (29.90)	9,708 (18.89)	9,048 (21.65)	384 (12.90)	4,038 (18.73)
90+	7,317 (4.03)	4,578 (7.15)	1,141 (2.22)	1,082 (2.59)	42 (1.41)	474 (2.20)
**Ethnicity**						
White	168,809 (92.87)	58,809 (91.82)	48,528 (94.41)	38,695 (92.60)	2,745 (92.24)	20,032 (92.93)
Other Ethnic Group[6]	6,256 (3.44)	2,300 (3.59)	1,396 (2.72)	1,568 (3.75)	111 (3.73)	881 (4.09)
Unknown[7]	6,698 (3.69)	2,936 (4.58)	1,475 (2.87)	1,525 (3.65)	120 (4.03)	642 (2.98)

Supplementary Table S2. Continued.

Analysis 1: Emergency Presentation		Emergency[1]	All Primary Care-Initiated Routes[2]			
**Analysis 2: Referral Urgency**				**Standard Primary Care-Initiated Routes[4]**		
	**Overall**	**Emergency**	**2WW[3]**	**Standard GP Referral**	**Inpatient Referral**	**Other Outpatient Referral**
	(n=181,763; 100%)	(n= 64,045; 35.24%)	(n=51,399; 28.28%)	(n=41,788; 22.99%)	(n= 2,976; 1.64%)	(n=21,555; 11.86%)
**Rural/Urban Residence**						
Rural Village, Hamlet & Isolated Dwellings	14,231 (7.83)	4,478 (6.99)	4,361 (8.48)	3,387 (8.11)	269 (9.04)	1,736 (8.05)
Rural Town & Fringe	17,439 (9.59)	5,920 (9.24)	5,248 (10.21)	4,085 (9.78)	274 (9.21)	1,912 (8.87)
Urban City & Town	80,331 (44.20)	27,978 (43.68)	24,321 (47.32)	17,727 (42.42)	1,498 (50.34)	8,807 (40.86)
Extensive Urban Area	69,762 (38.38)	25,669 (40.08)	17,469 (33.99)	16,589 (39.70)	935 (31.42)	9,100 (42.22)
**Government Region**						
North West	30,903 (17.00)	10,395 (16.23)	9,687 (18.85)	6,548 (15.67)	458 (15.39)	3,815 (17.70)
North East	13,301 (7.32)	4,640 (7.24)	4,002 (7.79)	2,972 (7.11)	228 (7.66)	1,459 (6.77)
West Midlands	18,661 (10.27)	6,642 (10.37)	4,513 (8.78)	4,757 (11.38)	246 (8.27)	2,503 (11.61)
Yorkshire & the Humber	21,535 (11.85)	7,803 (12.18)	6,307 (12.27)	4,946 (11.84)	267 (8.97)	2,212 (10.26)
East Midlands	15,654 (8.61)	5,559 (8.68)	5,054 (9.83)	3,390 (8.11)	222 (7.46)	1,429 (6.63)
East of England	18,765 (10.32)	6,386 (9.97)	5,120 (9.96)	4,647 (11.12)	397 (13.34)	2,215 (10.28)
South East	26,027 (14.32)	8,892 (13.88)	6,807 (13.24)	6,282 (15.03)	603 (20.26)	3,443 (15.97)
South West	17,738 (9.76)	6,091 (9.51)	5,726 (11.14)	3,892 (9.31)	285 (9.58)	1,744 (8.09)
London	19,179 (10.55)	7,637 (11.92)	4,183 (8.14)	4,354 (10.42)	270 (9.07)	2,735 (12.69)
**Stage at Diagnosis**						
I	27,476 (15.12)	4,898 (7.65)	7,387 (14.37)	9,303 (22.26)	246 (8.27)	5,642 (26.17)
II	13,520 (7.44)	2,557 (3.99)	4,981 (9.69)	3,765 (9.01)	140 (4.70)	2,077 (9.64)
III	35,303 (19.42)	8,071 (12.60)	14,009 (27.26)	8,411 (20.13)	637 (21.40)	4,175 (19.37)
IV	88,363 (48.61)	39,320 (61.39)	23,466 (45.65)	16,315 (39.04)	1,716 (57.66)	7,546 (35.01)
Unknown[8]	17,101 (9.41)	9,199 (14.36)	1,556 (3.03)	3,994 (9.56)	237 (7.96)	2,115 (9.81)

Supplementary Table S2. Continued.

Analysis 1: Emergency Presentation	Emergency[1]	All Primary Care-Initiated Routes[2]			
**Analysis 2: Referral Urgency**			**Standard Primary Care-Initiated Routes[4]**		
**Overall** (n=181,763; 100%)	**Emergency** (n= 64,045; 35.24%)	**2WW[3]** (n=51,399; 28.28%)	**Standard GP Referral** (n=41,788; 22.99%)	**Inpatient Referral** (n= 2,976; 1.64%)	**Other Outpatient Referral** (n=21,555; 11.86%)
**Histology**					
SCLC 19,125 (10.52)	6,565 (10.25)	6,366 (12.39)	3,670 (8.78)	572 (19.22)	1,952 (9.06)
NSCLC[9] 157,214 (86.49)	56,333 (87.96)	43,579 (84.79)	36,436 (87.19)	2,284 (76.75)	18,582 (86.21)
Other[10] 5,424 (2.98)	1,147 (1.79)	1,454 (2.83)	1,682 (4.03)	120 (4.03)	1,021 (4.74)
**Multiple Tumors[11]**					
No 149,148 (82.06)	54,736 (85.46)	43,316 (84.27)	32,549 (77.89)	2,586 (86.90)	15,961 (74.05)
Yes 32,615 (17.94)	9,309 (14.54)	8,083 (15.73)	9,239 (22.11)	390 (13.10)	5,594 (25.95)
**Number of Comorbidities[12]**					
0 101,797 (56.01)	33,256 (51.93)	33,451 (65.08)	22,172 (53.06)	2,078 (69.83)	10,840 (50.29)
1-2 56,059 (30.84)	20,524 (32.05)	13,795 (26.84)	13,793 (33.01)	698 (23.45)	7,249 (33.63)
3+ 23,907 (13.15)	10,265 (16.03)	4,153 (8.08)	5,823 (13.93)	200 (6.72)	3,466 (16.08)
**Discussed at MDT**					
Yes 83,489 (45.93)	25,954 (40.52)	31,261 (60.82)	16,032 (38.37)	1,367 (45.93)	8,875 (41.17)
No 30,631 (16.85)	8,490 (13.26)	10,328 (20.09)	7,158 (17.13)	489 (16.43)	4,166 (19.33)
Missing 67,643 (37.21)	29,601 (46.22)	9,810 (19.09)	18,598 (44.51)	1,120 (37.63)	8,514 (39.50)
**Diagnosis Year**					
2012 36,067 (19.84)	13,124 (20.49)	10,322 (20.08)	7,910 (18.93)	534 (17.94)	4,177 (19.38)
2013 36,157 (19.89)	13,087 (20.43)	10,112 (19.67)	8,042 (19.24)	601 (20.19)	4,315 (20.02)
2014 36,506 (20.08)	12,992 (20.29)	10,176 (19.80)	8,532 (20.42)	637 (21.40)	4,169 (19.34)
2015 36,516 (20.09)	12,616 (19.70)	10,402 (20.24)	8,652 (20.70)	622 (20.90)	4,224 (19.60)
2016 36,517 (20.09)	12,226 (19.09)	10,387 (20.21)	8,652 (20.70)	582 (19.56)	4,670 (21.67)

[1]Emergency refers to A&E, emergency GP referral, emergency transfer, and emergency admission or attendance. [2]All primary care-initiated routes refers to standard GP referral, inpatient elective, outpatient (other) and 2WW (urgent care referral via GP). [3]2WW refers to GP referral via the Urgent Care Pathway. [4]Standard primary care-initiated Routes refers to standard GP referral, inpatient referral, and other outpatient referral. [5]Refers to IMD (income domain). For diagnosis year 2012, IMD_2010 was used and for diagnosis years 2013-2016, IMD_2015 was used. [6]Other ethnic group refers to Asian/British Asian, Black/African/Caribbean/Black British, mixed/multiple ethnic groups and other ethnic groups. [7]Unknown ethnicity refers to unknown and missing ethnicity classifications. [8]Unknown staging refers to missing and unstageable tumors.

[9]NSCLC refers to adenocarcinomas, squamous cell, large cell, and not otherwise specified NSCLC. [10]Other histology refers to other specified and non-specified lung cancers. [11]Refers to any tumor(s) other than the index lung cancer. Yes, indicates that the patient had previously (i.e. before the current lung cancer diagnosis) been diagnosed with another cancer. [12]Refers to number of comorbidities between 78 to 6 months prior to diagnosis as determined by the Charlson Comorbidity Index. Abbreviations: IMD: Index of Multiple Deprivation (income domain); MDT: Multi-disciplinary Team; NSCLC: Non-small cell lung cancer; SCLC: Small cell lung cancer; 2WW: Two week wait.

Supplementary Table S3. Unadjusted analyses of associations between socio-demographic variables and route to diagnosis for patients with lung cancer diagnosed between 01/02/2012-31/12/2016: Likelihood of (i) emergency presentation (diagnosis via the emergency route versus all primary care-initiated routes, *n*=181,763) and (ii) referral urgency (diagnosis via the 2WW pathway (urgent referral via GP) vs all other standard primary care-initiated routes, *n*=117,718). Unadjusted odds ratios (OR) with 95% confidence intervals.

	**Analysis 1: Emergency presentation vs all primary care-initiated routes**[1]		**Analysis 2: 2WW pathway vs all other standard primary care-initiated routes**[2]	
	OR	***95% CI***	***OR***	***95% CI***
**Deprivation**[3]				
1 (Least Deprived)	1.00	-	1.00	-
2	1.06	1.02 – 1.10	1.08	1.04 – 1.13
3	1.13	1.10 – 1.17	1.07	1.02 – 1.11
4	1.20	1.16 – 1.24	1.09	1.05 – 1.13
5 (Most Deprived)	1.22	1.18 – 1.26	1.04	1.00 – 1.08
**Sex**				
Male	1.00	-	1.00	-
Female	1.04	1.02 – 1.06	1.01	0.98 – 1.03
**Age at Diagnosis (Years)**				
<50	0.97	0.91 – 1.03	0.89	0.83 – 0.96
50 – 59	0.86	0.83 – 0.89	1.09	1.05 – 1.14
60 – 69	0.82	0.80 – 0.84	1.11	1.07 – 1.14
70 – 79	1.00	-	1.00	-
80 – 89	1.72	1.67 – 1.76	0.95	0.92 – 0.98
90+	3.47	3.30 – 3.65	0.94	0.87 – 1.02
**Ethnicity**				
White	1.00	-	1.00	-
Other Ethnic Group[4]	1.09	1.03 – 1.15	0.69	0.65 – 0.74
Unknown[5]	1.46	1.39 – 1.53	0.82	0.76 – 0.87
**Rural/Urban Residence**				
Rural Village, Hamlet & Isolated Dwellings	0.79	0.76 – 0.82	1.23	1.18 – 1.29
Rural Town & Fringe	0.88	0.85 – 0.91	1.28	1.22 – 1.33
Urban City & Town	0.92	0.90 – 0.94	1.32	1.29 – 1.36
Extensive Urban Area	1.00	-	1.00	-
**Government Region**				
North West	1.00	-	-	-
North East	1.06	1.01 – 1.10	-	-
West Midlands	1.09	1.05 – 1.13	-	-
Yorkshire & the Humber	1.12	1.08 – 1.16	-	-
East Midlands	1.09	1.04 – 1.13	-	-
East of England	1.02	0.98 – 1.06	-	-
South East	1.02	0.99 – 1.06	-	-
South West	1.03	0.99 – 1.07	-	-
London	1.31	1.26 – 1.36	-	-

[1]Emergency refers to A&E, emergency GP referral, emergency transfer and emergency admission or attendance. [2]All primary care-initiated routes refers to standard GP referral, inpatient referral, outpatient (other) referral and 2WW (urgent care referral via GP). [3]Refers to IMD (income domain). For diagnosis year 2012, IMD_2010 was used and for diagnosis years 2013 - 2016, IMD_2015 was used. [4]Other ethnic group refers to Asian/British Asian, Black/African/Caribbean/Black British, mixed/multiple ethnic groups and other ethnic groups. [5]Unknown ethnicity refers to missing and unknown ethnicity classifications. Abbreviations: IMD: Index of Multiple Deprivation; mvOR: multivariable odds ratio; 95% CI: 95% Confidence interval. 2WW: Two week wait.
